# A decision support system for osteoporosis risk prediction using machine learning and explainable artificial intelligence

**DOI:** 10.1016/j.heliyon.2023.e22456

**Published:** 2023-12-02

**Authors:** Varada Vivek Khanna, Krishnaraj Chadaga, Niranjana Sampathila, Rajagopala Chadaga, Srikanth Prabhu, Swathi K S, Aditya S. Jagdale, Devadas Bhat

**Affiliations:** a Department of Biomedical Engineering, Manipal Institute of Technology, Manipal Academy of Higher Education, India; b Department of Computer Science and Engineering, Manipal Institute of Technology, Manipal Academy of Higher Education, India; c Department of Mechanical and Industrial Engineering, Manipal Institute of Technology, Manipal Academy of Higher Education, India; d Department of Social And Health Innovation, Prasanna School of Public Health, Manipal Academy of Higher Education, Manipal, 576104, India; e Mahatma Gandhi Institute of Medical Sciences, Sevagram, Maharashtra, India

**Keywords:** Ensemble-learning, Explainable machine learning, Feature selection techniques, Machine learning, Osteoporosis

## Abstract

Osteoporosis is a metabolic bone condition that occurs when bone mineral density and mass decrease. This makes the bones weak and brittle. The disorder is often undiagnosed and untreated due to its asymptomatic nature until the manifestation of a fracture. Machine Learning (ML) is extensively used in diverse healthcare domains to analyze precise outcomes, provide timely risk scores, and allocate resources. Hence, we have designed multiple heterogeneous machine-learning frameworks to predict the risk of Osteoporosis. An open-source dataset of 1493 patients containing bone density, blood, and physical tests is utilized. Thirteen distinct feature selection techniques were leveraged to extract the most salient parameters. The best-performing pipeline consisted of a Forward Feature Selection algorithm followed by a custom multi-level ensemble learning-based stack, which achieved an accuracy of 89 %. Deploying a layer of explainable artificial intelligence using tools such as SHAP (SHapley Additive Values), LIME (Local Interpretable Model Explainer), ELI5, Qlattice, and feature importance provided interpretability and rationale behind classifier prediction. With this study, we aim to provide the holistic risk prediction of Osteoporosis and concurrently present a system for automated screening to assist physicians in making diagnostic decisions.

## 1 Introduction

**Fig. 1:**
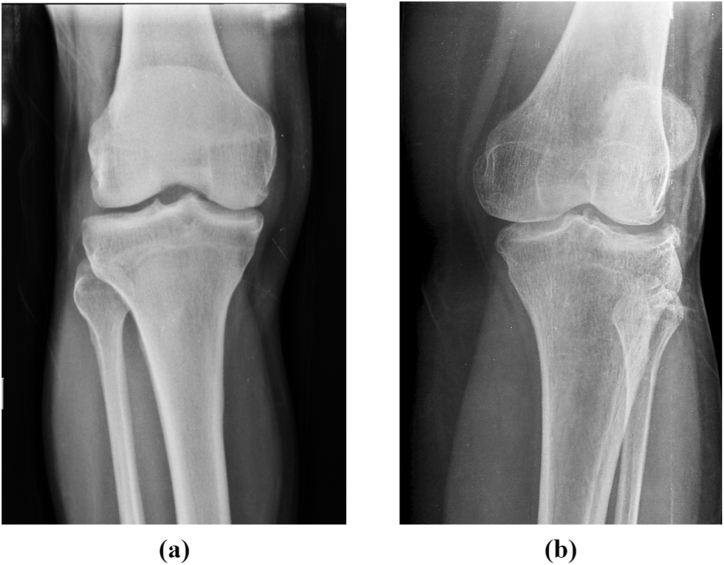
Open-source Knee-Xray scans: (a) Depict the normal patient, (b) Depicts the scan of an Osteoporosis patient (Steven [2]).

Osteoporosis (OP) is a prevalent condition that causes fragility fractures due to a systemic deterioration of bone mass and microarchitectures. Bone remodeling is a lifelong process entailing a continuous cycle of bone resorption and formation. One in three women and one in five men over the age of 50 are affected by osteoporosis worldwide [1]. The mature or damaged bone is resorbed during the resorption phase, transferring calcium and minerals from bone tissue to the blood. In bone formation, osteoblasts form new bone material until the reabsorbed bone is replaced (Parfitt [2]). Fig. 1 depicts the X-ray scans of a healthy individual in 1(a) and an osteoporosis patient in 1(b) (Steven [3]). For the OP-positive patient, the X-rays are not obstructed by dense bone structures, appearing grey rather than whiter. When reabsorption is greater than bone formation, the bone mass is reduced, and the bone becomes more porous, eventually leading to a higher fracture risk. Low estrogen, low serum calcium levels, alcohol consumption, and smoking increase the risk of osteoporosis (Rachner, Khosla, and Hofbauer [4]). Diseases such as Diabetes Mellitus, Hypertension, Hyperlipidemia, and Hyperuricemia have been linked to accelerating osteoporosis (Leidig-Bruckner, G., and R. Ziegler. [5], Parhami [6], Ilić, K., Obradović, N., & Vujasinović-Stupar, N [7]. and Lin et al. [8]). Osteoporosis is often called a "silent" disease as this disorder is typically asymptomatic until bone fracture (Ralston and Fraser [9]). This condition is primarily diagnosed by assessing bone density using Dual-energy X-Ray Absorptiometry (DXA) Scan. The results of the DXA scan are produced by comparing the patient's bone density with a normal adult, yielding a T-score. A T-score less than or equal to −2.5 is diagnostic of osteoporosis (Mafi Golchin et al. [10]). The T-score, blood parameters, and the prevalence of a particular disease could help predict the holistic risk of osteoporosis patients.

Osteoporosis is considered one of the serious public health issues. Even though specific treatments can reduce fracture risk by 33 %–50 % (Hochberg [11]), only a small percentage of OP patients who have previously suffered osteoporotic fractures get the proper diagnosis and care. Assessing the risk of osteoporosis is required to improve clinical decision-making and healthcare management. Machine Learning (ML) and Deep Learning (DL) applications are widely deployed towards improving healthcare diagnosis for OP patients for screening, risk assessment, and prognosis (Smets et al. [12]). ML-based decision support systems are revolutionizing several domains of life sciences and environmental sciences **(Gheibi et al.** [13]**, Ghadami et al.** [14]**, Pouresmaeil et al.** [15] **and Ghazikhani et al.** [16]**).** These advancements in ML and DL techniques can effectively detect patterns in bio-signals and images to produce predictive analytics for a patient in the domain of medical sciences. Advancement in healthcare artificial intelligence (AI) has led to several developments in automating diagnosis, patient-triaging, risk prediction, drug delivery, and deployment of decision support systems (Khanna et al. [17], Chadaga et al. [18]).

Multiple ML and DL models have been suggested to detect and assess the risk of OP. Shim et al. [19] deployed K-nearest Neighbors (KNN), Decision Tree (DT), Random Forest (RF), Logistic Regression (LR), Gradient Boosting Machine (GBM), Support Vector Machine (SVM), and Artificial Neural Networks (ANN) to predict the risk of OP. A total of 613 Korean postmenopausal women had OP. ANN trained on nine selected features provided the best performance with an Area Under the Receiver Operating Curve score (AUROC) of 0.743. Yoo et al. [20] trained ML classifiers for the Korean postmenopausal women dataset. Various wrapper-based and embedded methods of feature selection techniques were explored for different ML classifiers. With the wrapper-based method for feature extraction, SVM outperformed ANN, LR, and RF with an AUROC score of 0.827 and an accuracy of 77.8 %. Kwon et al. [21] built an ensemble machine-learning model for screening OP among postmenopausal Korean women. Data from 1431 patients were selected, followed by extracting 20 features by feature importance and recursive feature elimination. Three tree-based models, RF, AdaBoost, and Gradient Boosting were trained, and AdaBoost achieved an accuracy of 82.90 % and an AUROC score of 0.921. Erjiang et al. [22] aimed to propose algorithms to assess the risk of fractures, improving DXA predictions and providing accurate diagnosis OP. A cohort of 13,577 patients aged 40 and above was selected. Extreme gradient boosting algorithm outperformed CatBoost, Neural network, bagged flexible discriminant analysis, RF, LR, and SVM, achieving an AUROC score of 0.723 and 0.810 for classifying OP among older men and women, respectively. Ou Yang et al. [23] considered 5982 patients in Taiwan. The Hematological and Biochemical profiles, along with DXA-based T-scores, were considered. They constructed different OP detection architectures for men and women using ANN. SVM. RF, KNN, and LR. The best-performing algorithm for men was SVM, with an AUROC score of 0.837; for women, RF achieved the highest score of 0.811. Bui et al. [24] explored four models, namely, LR, SVM (polynomial kernel), RF, and ANN (with adam and stochastic gradient optimizer), to predict the risk of OP among a Vietnamese population. The highest AUROC score obtained was 0.862 for RF.

The motivation of this study is to design and evaluate multiple ML classifiers to assess a patient's OP risk accurately. Feature engineering is a vast domain. If the optimal and correct features are selected, the performance of the classifiers can immensely improve. The pipelines were constructed with 13 feature selection methods and 12 ML models. Further, a customized multi-level stack pipeline is proposed. Explainable Machine Learning (XML) is being extensively utilized to provide predictions made by traditional Black box classifiers for deeper understanding and interpretability. It is essential for a domain expert to demand a detailed rationale about individual classifications, particularly when making life-altering decisions for the patient's well-being. Shapley Additive Values (SHAP), Local Interpretable Model Explainer (LIME), ELI5, Qlattice, and Feature Importance were used in this research to provide better explainability.

The main contributions of this research are as follows.

• Comparison of thirteen feature selection techniques as Mutual Information, Pearson Correlation, Recursive Feature Elimination, Forward Feature Selection, Harris Hawk Optimization, Grey Wolf Optimizer, Whale Optimization, Salp Swarm optimization, Bat optimization, Cuckoo Search optimization, Firefly algorithm, Flower Pollination optimization, and Particle Swarm optimization.
• Analyze classifier performance of eleven ML models such as LR, Decision Trees, RF, SVM (with Linear, Polynomial, sigmoidal, and gaussian kernels), Naïve Bayes, KNN, AdaBoost, XGBoost, Extratrees, Light GBM, and Catboost. A multi-level custom ensemble stacking pipeline with the parallel sequence of the models mentioned above was further developed.
• Deployment of XML tools such as SHAP, LIME ELI5, and Qlattice. Comparing these XAI techniques with Feature importance for tree-based models.

The succeeding sections are section 2, which contains Materials and Methodology. This section gives insights into the data selected, processing, feature engineering techniques, and the creation of ML pipelines. Section 3 presents the results obtained for different architectures and the deployment of XML for the best architecture. Our study is compared with similar existing studies and discusses the significant results. The final section concludes the study and presents the future vision of this research.

## 2 Methods and methodology

This section explores various techniques and algorithms for data engineering and building ML pipelines. Our research was conducted on Python 3.8.10 with an NVIDIA GeForce GTX 1050 Ti- GPU system. Python libraries like Pandas, NumPy, SciPy, and Scikit-learn were used for data engineering, visualizations, and creating machine learning classifiers.

### 2.1 Data description

This research utilized the 'Bone Mineral Density' open-source dataset accessible on Harvard Dataverse, published by **He, Linfeng** [25]. The original dataset had 1537 observations and 40 variables. The target variable for this dataset is 'OP'. Six of the 39 remaining features were bone density and DXA T-scores for lumbar 1–4 (L1-4), Femoral Neck (FN), and Thoracolumbar (TL) bones. Eleven features consisted of the patient's hematological and biochemical profiles. The renal profile indicators were Uric acid (URIC), Creatinine (CREA), Calcium (Ca), Phosphorous (P), Magnesium (Mg), and Blood Urea Nitrogen (BUN). The hepatic panel consisted of Alanine Transaminase (ALT) and Aspartate Aminotransferase (AST) values. The lipid profile was assessed with High-Density Lipoprotein Cholesterol (HDL-C) and Low-Density Lipoprotein Cholesterol (LDL-C). FBG indicated Fasting Blood sugar. Four features determined the administration of drugs such as Bisphosphonate, Calcitonin, Calcitriol, and Calcium. Eleven features encompassed whether the patient faced other Osteoporosis-related disorders. Two features were dedicated to obtaining data about the patient's smoking and drinking habits. Five parameters indicated gender, age, height, weight, and Body Mass Index (BMI).

### 2.2 Data pre-processing

**Fig. 2:**
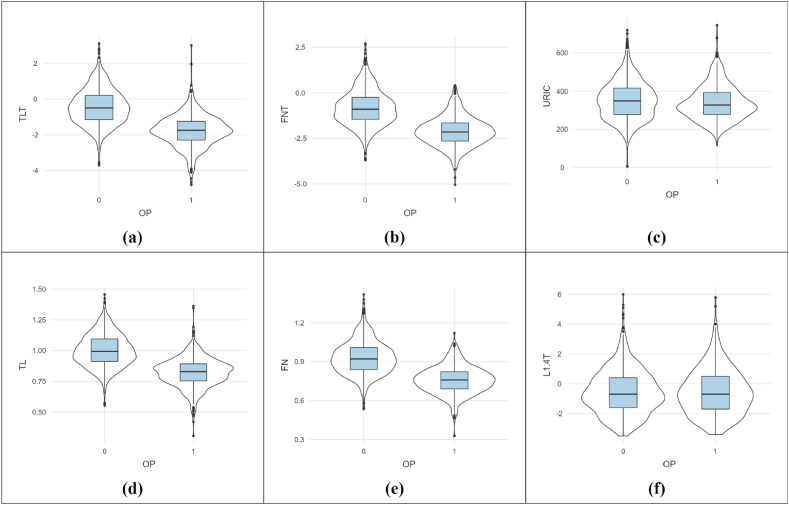
Violin and Box plots of some numerical features. The following plots indicate: (a) the TLT DXA score, (b) the FNT DXA score, (c) URIC acid values, (d) TL bone density values, (e) FN bone density values and (f) L1-4 DXA score for normal and OP-positive patients.

During data pre-processing, we observed that the data had multiple missing values. Attributes such as age, height, weight, BMI, HDL-C, LDL-C, P, Mg, Ca, ALT, and AST had missing data. The missing values were replaced with the feature median, as the feature's statistical mean can be severely affected by outliers (Leys et al. [26]). Lack of appropriate metadata led to removal of the features VD, VT, AS, followed by drug administration data variables such as Bisphosphonate, Calcitonin, Calcitriol, and Calcium. We dropped the 'Gender' parameter due to unidentified indexing. The dataset now had 1492 samples and 30 variables. Parameters such as HTN (Hypertension), COPD (Chronic obstructive pulmonary disease), DM (Diabetes Mellitus), Hyperlipidemia, Hyperuricemia, CAD (Coronary Artery Disease), CKD (Chronic Kidney Disease), and fracture were categorical variables with 0 and 1 coding. The value '1′ indicated the patient had a specific OP-related disorder. Additionally, this dataset has considered categorical variables like smoking and drinking habits. Table 1 describes the descriptive statistical significance of the parameters. Fig. 2 represents the violin and box plots of some numerical variables. Fig. 2(a) and (b) represent the DXA T-scores for the Thoracolumbar and Femoral neck scans, respectively. Both the violin and plots for these variables have significant variations for osteoporosis patients. As the literature suggests, T-scores less than or equal to −2.5 are categorized as OP patients (Parfitt [2]). The same can be observed in the first two plots. In Fig. 2(c), the shape of the serum uric acid violin plot for the normal and OP-positive patients is drastically different, indicating a correlation of the parameter with OP prediction. In Fig. 2(d) and (e), TL and FN bone densities are mapped, respectively. For osteoporosis patients, the bone density is lower than normal, depicted in Fig. 2(d) and (e). However, Fig. 2(f) depicts the similarities in DXA t-scores for the lumbar 1–4 bones, indicating no drastic observance of change between the OP-positive and normal cases. The conclusion of L1-4T not being a reliable variable for predicting the risk of OP can be made using the data.

**Table 1:** Descriptive statistics of dataset attributes.

Sr. No.	Attributes	Missing Values	Data Type	Mean	Std	Min	25 %	50 %	75 %	Max
1	Age	36	Numerical	59.85	12.93	28.6	51.00	57.00	67.28	99.8
2	Height	34	Numerical	125.82	8.09	141	160	167	172	187
3	Weight	34	Numerical	67.15	12	23	59	67	75	113
4	BMI	34	Numerical	24.31	3.32	9.21	22.07	24.22	26.37	37.26
5	L1-4	0	Numerical	1.14	0.19	0.77	1.00	1.13	1.26	1.94
6	L1.4T	0	Numerical	−0.55	1.52	−3.50	−1.60	−0.70	0.40	6.00
7	FN	0	Numerical	0.87	0.15	0.33	0.77	0.86	0.97	1.42
8	FNT	0	Numerical	−1.30	1.12	−5.05	−2.10	−1.40	−0.55	2.70
9	TL	0	Numerical	0.93	0.15	0.31	0.84	0.93	1.04	1.46
10	TLT	0	Numerical	−0.93	1.16	−4.80	−1.70	−1.00	−0.20	3.10
11	ALT	2	Numerical	23.44	16.53	4.00	14.00	19.00	28.00	181.00
12	AST	2	Numerical	22.60	9.34	9.00	17.00	21.00	25.00	128.00
13	BUN	1	Numerical	5.62	3.36	1.74	4.34	5.18	6.20	69.80
14	CREA	3	Numerical	74.09	25.95	5.86	60.00	70.00	81.80	381.20
15	URIC	0	Numerical	348.18	96.78	5.46	277.97	339.55	408.93	745.30
16	FBG	16	Numerical	5.33	1.55	3.13	4.59	4.97	5.53	24.65
17	HDL-C	17	Numerical	1.25	0.38	0.45	1.01	1.19	1.44	5.46
18	LDL-C	14	Numerical	2.60	0.90	0.14	1.92	2.55	3.17	6.65
19	Ca	2	Numerical	2.24	0.16	1.78	2.16	2.23	2.31	5.84
20	P	5	Numerical	1.04	0.21	0.56	0.92	1.02	1.13	4.41
21	Mg	3	Numerical	0.87	0.10	0.09	0.81	0.87	0.93	1.73
22	HTN	0	Categorical	NA	NA	0	NA	NA	NA	1
23	COPD	0	Categorical	NA	NA	0	NA	NA	NA	1
24	DM	0	Categorical	NA	NA	0	NA	NA	NA	1
25	Hyperlipidemia	0	Categorical	NA	NA	0	NA	NA	NA	1
26	Hyperuricemia	0	Categorical	NA	NA	0	NA	NA	NA	1
27	CAD	0	Categorical	NA	NA	0	NA	NA	NA	1
28	CKD	0	Categorical	NA	NA	0	NA	NA	NA	1
29	Fracture	0	Categorical	NA	NA	0	NA	NA	NA	1
30	Smoking	0	Categorical	NA	NA	0	NA	NA	NA	1
31	Drinking	0	Categorical	NA	NA	0	NA	NA	NA	1
32	OP	0	Categorical	NA	NA	0	NA	NA	NA	1

Further, the data was split into training and testing sets by the 80:20 data ratio. 80 % of the data would train our pipelines, and 20 % would be used to evaluate our architecture. Feature scaling followed this train-test split. Standardization is rescaling the feature values to fall between 0 and 1. This is exceptionally helpful in the optimization of algorithms. Compared with the min-max scalar, data scaled by standard scalar makes the algorithms less sensitive to outliers (Ferreira, P., Le, D. C., & Zincir-Heywood, N [27]). Hence, a standard scalar was used in this study.

553 out of the 1492 patients were diagnosed with OP, indicating a skew in the classification class. This imbalance leads to the risk of the classifier only predicting the majority class. This could cause higher false negative predictions (Longadge and Dongre [28]). To alleviate the data imbalance, we deployed the Borderline Synthetic Minority Oversampling Technique (SMOTE) (Han, H., Wang, W. Y., & Mao, B. H. [29]). As its name suggests, SMOTE creates synthetic data samples in the minority class to match the majority class. Misclassification of outliers is significantly lower in Borderline SMOTE compared to traditional SMOTE. After balancing the training data with Borderline SMOTE, the count for positive and negative OP cases was 939.

### 2.3 Feature selection

**Fig. 3:**
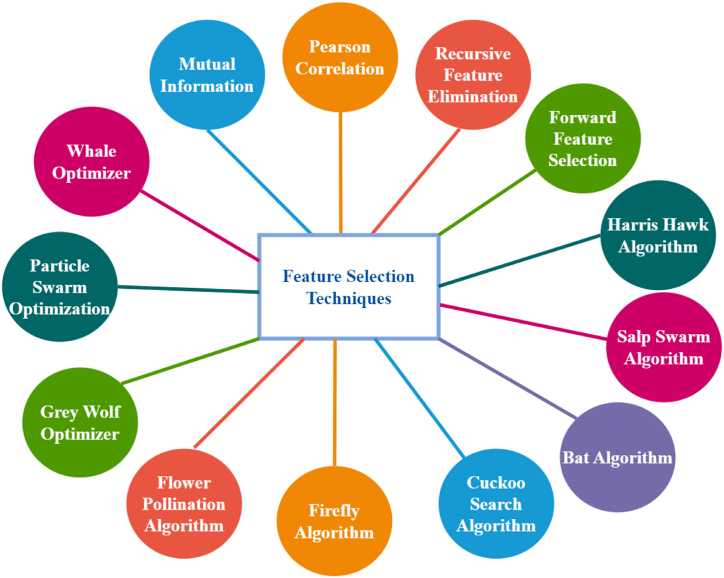
Feature selection techniques.

This section discusses thirteen distinctive feature extraction techniques deployed. Two filter methods and eleven wrapper methods are utilized in this study. Nine wrapper methods Feature Selection (FS) are meta-heuristic, nature-inspired algorithms. These algorithms assisted in extracting the most significant features by different processes. For all Nature-inspired algorithms, we used a ‘Feature Selection Wrapper Class’ algorithm from a GitHub toolbox **(Too, Jingwei** [30]**)**. Fig. 3 represents the feature selection techniques used in this study.

#### 2.3.1 Filter methods

**Fig. 4:**
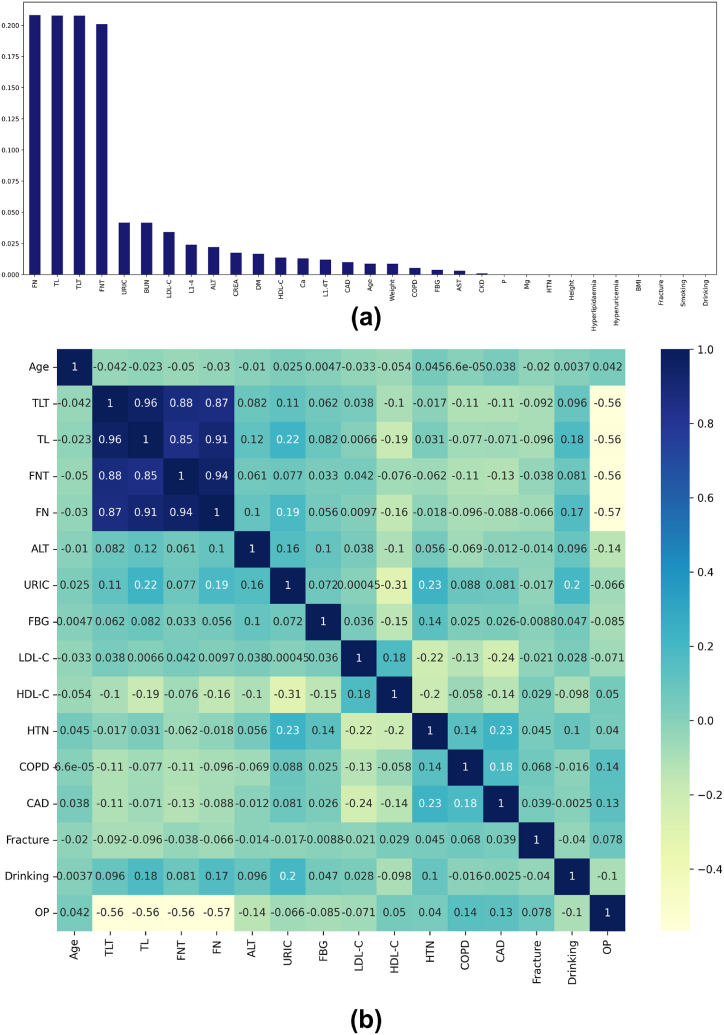
Filter method: (a) Mutual Information descending dependency graph, (b) Pearson Correlation Heatmap.

The Filter method is a feature selection technique independent of the classifier used. It measures the significance and relevance of the feature based on univariate statistics (Talavera [31]). These techniques are known to be faster and less computationally intensive than wrapper methods. Under filter algorithms, we have utilized the Mutual information (MI) and Pearson correlation (PC) techniques. It measures the relationship between individual features and the target. Mutual information measures the features' linear and non-linear dependence on the target (Steuer et al. [32]). Fig. 4(a) describes the features chosen by mutual information in the descending order of their importance. The top fifteen most significant features were manually extracted. All bone density and lipid profile parameters were selected, followed by the biomarker profiles and the presence of Diabetes Mellitus and Coronary Artery Disease.

Pearson Correlation estimates the strength of the linear relationship between two features (Jöreskog [33], Kohavi Ron [34]). While exploring this technique, the Bone density and DXA T-score parameters had a >0.5 correlation with the target. The biomarkers and OP-related disorders had a <0.5 correlation with the Osteoporosis diagnosis. As this study aims to provide a holistic risk prediction for OP, the threshold for feature correlation was set to 0.04. This threshold assisted extraction of 15 features depicting the biomarker profiles and Bone density information. Fig. 4(b) is a Pearson Heatmap indicating the correlation between the top selected 15 independent features with the target. The selected variables suggest that lipid profiles and the presence of Hypertension, Chronic obstructive pulmonary disease, and coronary artery disease can be the chief contributors to putting the patient at risk of OP, according to Pearson's correlation technique.

#### 2.3.2 Wrapper methods

Wrapper algorithms select 'useful' features that ensure improved and optimized classifier performance with repeated learning steps and cross-validation (Kohavi and Sommer [35]). In this subsection we aim to compare the performance of some widely used feature selection wrapper techniques for selecting the optimal number of features. The algorithms used are as follows.

• **Recursive Feature Elimination (RFE):** This technique calculates the importance of each feature at each stage and eliminates the least significant feature until the desired number of features remains (G**uyon, I., Weston, J., Barnhill, S., and Vapnik, V.** [35]**)** In our study, Logistic Regression estimates the feature importance. RFE is a widely used algorithm because of its ease to configure and efficiency in selecting relevant features. In this study, RFE selected 15 features. This method relates the presence of multiple disorders like DM, CAD, CKD, COPD, and Hyperlipidaemia to increasing the risk of osteoporosis.
• **Forward Feature Selection (FSE):** In contrast to RFE, Forward Feature Selection (FSE) starts with no features. The best feature that improves the model performance is added in each iteration until the desired number of features are obtained **(Weston et al.** [36]**).** A Linear Regression model calculates the feature importance of FSE. This method starts with smaller models and is less susceptible to high intercorrelation among the features. FSE chose 15 features consisting of Bone density T-scores, renal, hepatic, and lipid profile indicators, and disorder history.

Further, nine feature selection methods dedicated to the meta-heuristic nature-inspired wrapper methods were utilized. These methods have distinctive procedures for extracting the best features **(Yagiura and Ibaraki** [37]**).** Furthermore, the algorithm decides the number of best features. Swarm intelligence-based algorithms consider a population of unsophisticated agents representing a potential solution (Br**ezočnik, Lucija, Iztok Fister Jr, and Vili Podgorelec**. [38]**)**.

• **Harris Hawk Optimizer (HHO)**: This feature selection method mimics the behavior and collaborative strategy of Harris hawks while chasing the prey. This algorithm was proposed by **Heidari et al**. [39]. This swarm-based, gradient-free optimization and the evolutionary algorithm uses various stochastic components that determine the best parameters during the time-varying exploration and exploitation phases. This algorithm is known for its fast convergence and strong local search capabilities. HHO suggested 14 features that were manually extracted.
• **Grey Wolf Optimizer (GWO)**: GWO mimics grey wolves' leadership hierarchy and hunting behavior. This optimizer was proposed by **Mirjalili, S., Mirjalili, S. M., and Lewis, A** [40]**.** The leadership hierarchy is simulated by four types of wolves: alpha, beta, delta, and omega. The prey is tracked, encircled, and attacked by the grey wolves. The alpha wolf is the fittest solution that leads the hunting process, followed by betas, deltas, and omegas. The position of the pack members is a function of the position of the Alpha wolf and the target. GWO can perform superior to other algorithms owing to its strong global-optimization ability.
• **Whale Optimization (WO)**: The Humpback whales' hunting strategies have influenced the mathematical modeling of the Whale Optimizer (WO). **Mirjalili, S., and Lewis, A** [41]. developed the WO algorithm. It consists of three phases searching, encircling, and hunting. The whales entrap and shrink the prey group through bubble net feeding during target foraging. Once the best search agent is defined, the other search agent positions are updated until the optimum solution is not obtained. However, it tends to have low accuracy and convergence speed.
• **Salp Swarm Algorithm (SSA)**: This algorithm is mathematically modeled to represent the Salp chain behavior while forging the ocean for food. This method was designed by **Mirjalili et al**. [42]**.** The chain population is split into a leader and followers. The position of the followers is updated based on the position of the leader and the 'target'. SSA demonstrates swarm intelligence and the ability to solve issues of slow convergence rate. The method can avoid local optimums and successfully balance its exploration and exploitation phases.
• **Bat Algorithm (BA)**: This algorithm aims to mimic the echolocation behavior of microbats to catch prey. **Yang, Xin-She** [43]. proposed this metaheuristic algorithm. The echo that bounces back from the prey helps the bat locate its target. Each bat (each solution) is initially assigned a random velocity and position frequency value. With each iteration of this algorithm, these values are updated. The best solution is selected based on the higher frequency and lower loudness. BA entails obtaining solutions based on population and local search algorithms.
• **Firefly algorithm (FFA)**: Another efficient and easy-to-implement algorithm is the Firefly algorithm (FFA). The flashing behavior of fireflies inspires this meta-heuristic swarm-based algorithm. FFA was proposed by **Xin-She, Y., & Slowik, A.** [44]**.** In this algorithm, the fireflies are randomly generated solutions. The brightness of each depends on the performance of the objective function. This deterministic algorithm produces an optimal solution in a small number of iterations.
• **Particle Swarm Optimizer (PSO)**: This algorithm takes inspiration from a flock of birds randomly flying to search for food. PSO was proposed by **Eberhart, R., and Kennedy, J.** [45]**.** This nature-inspired algorithm is a population-based stochastic search algorithm that searches for the optimum values with multiple iterations. The best position for the birds is initially unknown. However, if any member finds a desirable path, all others follow. The velocity and position are updated with each iteration. This optimizer has efficient global search abilities, is easy to implement, and is derivative-free.
• **Flower Pollination Algorithm (FPA)**: This algorithm bio-mimics flowering species' cross and self-pollination process. FFA was developed by **Yang, X. S.** [46]**.** FPA is a meta-heuristic algorithm with both an exploration and exploitation phase for global and local pollination. A fitness function evaluates the performance of each search agent. This algorithm is known for its simplicity and effectiveness over PSO.
• **Cuckoo search algorithm (CSA)**: This algorithm is based on the brood parasitism of certain cuckoo species by laying their eggs in the nest of host birds. **Yang, X. S., and Deb, S** [47]. proposed CSA. They observed that if the host bird identifies the egg as an alien, it can throw the egg or abandon the nest. However, if it remains successfully camouflaged among other eggs, the host will feed and provide for the cuckoo bird as its own. Each egg represents a solution, and the cuckoo egg represents a better solution to replace the not-so-good solutions in the nest. This trajectory-based algorithm utilizes a stochastic process of levy flight, which is considered more effective than any other random-walk-based randomization technique.

**Fig. 5:**
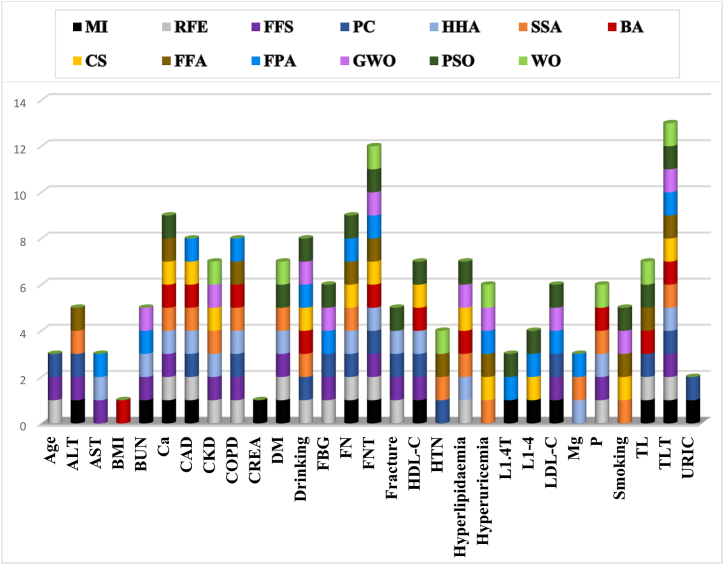
Parameters selected by 13 Feature Selection Techniques where MI: Mutual Information, RFE: Recursive Feature Elimination, FFS: Forward Feature Selection, HHO: Harris Hawk Optimization, SSA: Salp Swarm Algorithm, BA: Bat Algorithm, CS: Cuckoo Search, FFA: Firefly Algorithm, FPA: Flower Pollination Algorithm, GWO: Grey Wolf Algorithm, PSO: Particle Swarm Optimizer, WO: Whale Optimization.

Fig. 5 depicts the features selected by the 13 distinctive algorithms. Among the 31 independent variables, none of the algorithms extracted height and weight features, giving insights on these parameters not significantly affecting OP risk prediction. The 'CREA' variable was selected by no technique other than mutual information. Bone density DXA scores are considered the gold standard when predicting OP. This is reflected by all algorithms deployed, as most of them selected the TLT and FNT values to assess osteoporosis.

### 2.4 Machine learning models

After successfully extracting the features, the training data was used to train various Machine Learning classifiers. In our study, we have explored multiple ML models. Both linear and non-linear ML models were considered for this study. In recent studies various tree based bagging and boosting classifiers have been seen to outperform traditional ML models and neural networks. Sub-classifiers, namely: Logistic Regression (10.13039/501100009319LR), Decision Tree (DT), Random Forest (RF), Support Vector Machine (SVM), Naïve Bayes (NB), K-Nearest Neighbors (KNN), AdaBoost, Extratrees, Light Gradient Boosting Machine, Catboost. The Polynomial, Gaussian, Sigmoidal, and Linear Kernels of SVM were considered. GridSearchCV tuned the hyperparameters for all mentioned models. This algorithm performed an exhaustive search to explore all combinations of classifier parameters. It then predicts the optimum parameters leading to the best model performance.

**Fig. 6:**
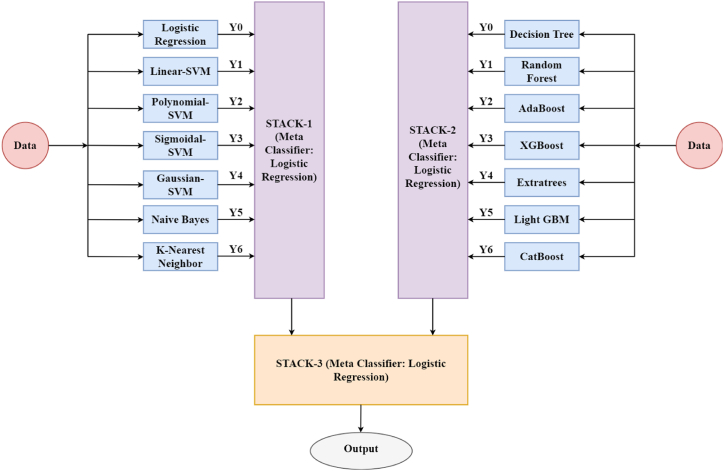
Pictorial representation of our customized Stack.

Model stacking is an ensemble learning approach that combines outputs of multiple models and processes these through a meta-learner. The meta-learners aim to minimize the weakness of individual sub-classifiers and maximize the strengths of every classifier. This stacking method then produces a superior and robust model. Three ensemble stacks were designed and developed. STACK-1 is a parallel sequence of LR, Linear-SVM, Polynomial-SVM, Gaussian-SVM, Sigmoidal-SVM, NB, and KNN. STACK-2 was a ‘super-tree-ensemble’, aggregating outputs of all the tree-based classifiers: DT, RF, AdaBoost, XGBoost, Extratrees, Light GBM, and CatBoost. STACK-3 was a multi-level ensemble of STACK-1 and STACK-2. All stacks have deployed Logistic Regression as their meta-classifier. Fig. 6 represents a visualization of the design of the customized stack.

**Fig. 7:**
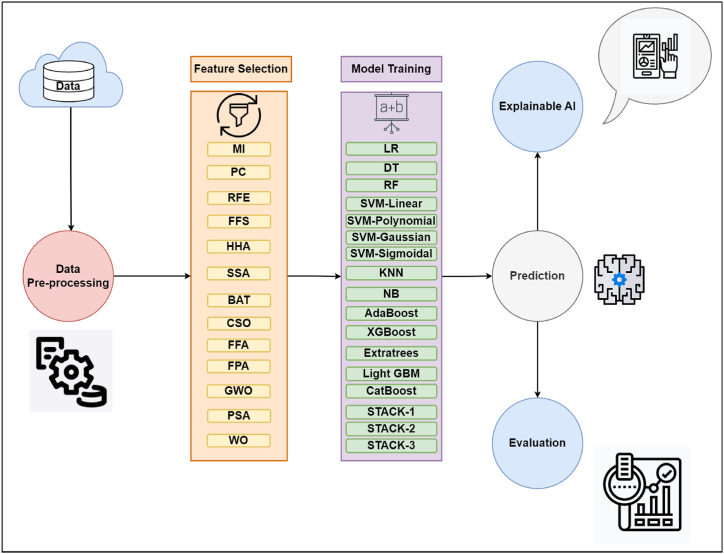
Process flow of machine learning for osteoporosis risk prediction.

Further, we deployed a layer of explainable AI to decode predictions made by the tree-based classifiers. Most tree-based models are a ‘black box’ where specific inputs will predict outputs without the user knowing the rationale of why the classifier made a prediction. We used tools like SHAP, LIME, ELI5, QLattice, and Feature Importances for the tree-based models to interpret the results. SHAP is a game-theory-based model that explains ML predictions by SHAP feature values that help produce visualizations. SHAP values estimate the impact of a feature on the predictions, whereas feature importance measures the impact of a feature on model fit. LIME explains individual patient-wise predictions and provides probabilistic risk evaluation for the patient. ELI5 tool is used to inspect and debug classifiers and provide insights into how a prediction was made. Qlattice creates an automated classification framework that produces simple equations and QGraphs to explain the created model **(Chadaga et al.** [48]**)**. Feature importance assigns a calculated value to each independent input feature of a model based on its relevance in predicting a target **(Linardatos, P., Papastefanopoulos, V., & Kotsiantis, S.** [49]**)** Feature importance in this study is used to obtain the reliability of the XAI techniques. An in-depth analysis of these techniques is portrayed in the result section. The overall model flow diagram is depicted in Fig. 7.

### 2.5 Performance metrics

After designing the pipelines, evaluation was conducted by various performance metrics. These are quantitative measures for accessing the predictive abilities of such ML pipelines.

Table 2 depicts the classification metrics, significance, and formulas used to evaluate the architectures. The classifier predictions are categorized into True Positive (TP), True Negative (TN), False Positive (FP), and False Negative (FN). These define metrics like accuracy, recall, precision, AUC-ROC score, and Mathew's correlation. A high-performing model will have accuracy, precision, recall, F-1 score, AUC-ROC score, Jaccard's score, and Mathew's correlation coefficient close to one. The log loss and hamming loss values are close to zero if the model is efficient **(Ferrer, L.** [50]**)**.

**Table 2:** Classification metric used in this study **(Ferrer, L. 2022)**.

Sr. No.	Classification metric	Definition	Formula
1	Accuracy	Measures number of all data points correctly classified by the algorithm.	(1) $Accuracy=\frac{TP+TN}{TP+TN+FP+FN}$
2	Precision	Measure of correctly predicted OP patients over all predictions made	(2) $Precision=\frac{TP}{TP+FP}$
3	Recall	Measure of actual positives that were incorrectly classified as OP-negative	(3) $Recall=\frac{TP}{TP+FN}$
4	F-1 Score	Harmonic mean of precision and recall	(4) $F-1\;score=2*\frac{Precision*Recall}{Precision+Recall}$
5	AUC-ROC score	Score obtained by the ROC graph that is plotted with True Positive rate against False Positive rate.	(5) $True\;Positive\;Rate=\frac{TP}{TP+FN}$ (6) $False\;Positive\;Rate=\frac{FP}{FP+TN}$
6	Hamming loss	Measure of fraction of data predicted incorrectly among all classifications made	(7) $$Hamming\;Loss=\frac{1}{nL}\sum_{i=1}^{n}\sum_{j=1}^{L}\left[I(y_{j}^{\left(i\right)}\neq {\hat{y}}_{j}^{\left(i\right)}\right]$$ Where, n = No. of training samples $y_{j}^{\left(i\right)}=True\;labels\;for\;the\;ith\;training\;samples\;in\;jth\;class$ ${\hat{y}}_{j}^{\left(i\right)}=Predicted\;labels\;for\;the\;ith\;training\;examples\;in\;jth\;class$
7	Jaccard Score	Measures the similarity between sample sets	(8) $Jaccard\;Score=J\left(A,B\right)=\frac{\left|A\cap B\right|}{\left|A\cup B\right|}$
8	Log Loss	Measure of closeness of prediction probability to the corresponding actual values.	(9) $H_{p}\left(q\right)=-\frac{1}{N}\sum_{i=1}^{N}y_{i}*\log\left(p\left(y_{i}\right)\right)+\left(1-y_{i}\right)*\log\left(1-p\left(y_{i}\right)\right)$
9	Mathew's correlation Coefficient	Measures the differences between predicted and actual values	(10) $MCC=\frac{TP*TN-FP*FN}{\sqrt{\left(TP+FP\right)\left(TP+FN\right)\left(TN+FP\right)\left(TN+FN\right)}}$

## 3 Results and discussion

In this section, we have evaluated and compared the performance of all ML pipelines created in this study. Explainable AI tools were deployed for the best-performing tree-based models, along with a comprehensive understanding of the feature importance.

The comparison of accuracies of all thirteen feature selection technique-trained models is presented in Table 3. It is observed that the classifiers trained on Forward Feature Selection engineered data obtained the best performance. Among the filter-method architectures, classifiers trained on PC-engineered data performed best. When considering the Nature-inspired meta-heuristic algorithms, GWO-data-trained classifiers outperformed all others. The lowest accuracy score is observed in the case of the Firefly pipeline, followed by the Whale Optimizer pipeline. HHO, SSA, BA, and CS architectures had similar average results. FPA and GWO frameworks scored better than the wrapper method RFE and the Filter Method MI. These results agree with research on FPA claiming to be better than Particle Swarm Algorithm **Eberhart, R., and Kennedy, J** [45]. Pearson Correlation and Flower Pollination algorithms-data trained the classifiers have produced comparable scores. The pipeline with Random Forest trained by FFS-data performed the best among all other sub-classifier architectures. This pipeline achieved an accuracy of 88 %. Tree-based models like RF, XGBoost, and Light GBM performed better than SVM, KNN, and NB throughout feature-engineered pipelines.

**Table 3:** Accuracy comparison of all ML models with different feature selection techniques.

Feature Selection Techniques	Filter Method		Wrapper										
					Nature inspired Meta-heuristic algorithm								
**Model**	**MI**	**PC**	**RFE**	**FFS**	**HHO**	**SSA**	**BA**	**CS**	**FFA**	**FPA**	**GWO**	**PSA**	**WO**
**Log Reg**	0.81	0.82	0.83	0.84	0.8	0.83	0.81	0.8	0.8	0.82	0.82	0.81	0.79
**Decision Tree**	0.81	0.81	0.77	0.8	0.78	0.77	0.79	0.74	0.85	0.78	0.79	0.77	0.78
**RF**	0.83	0.87	0.87	0.88	0.84	0.84	0.84	0.83	0.85	0.87	0.87	0.85	0.82
**SVM linear**	0.82	0.84	0.81	0.83	0.81	0.81	0.8	0.8	0.81	0.82	0.85	0.83	0.81
**SVM Poly**	0.81	0.81	0.8	0.83	0.82	0.8	0.75	0.8	0.78	0.79	0.76	0.81	0.79
**SVM gaussian**	0.83	0.84	0.82	0.84	0.81	0.81	0.81	0.81	0.79	0.81	0.84	0.8	0.82
**SVM sigmoid**	0.77	0.82	0.78	0.72	0.8	0.76	0.77	0.78	0.77	0.75	0.74	0.79	0.73
**Naïve bayes**	0.82	0.82	0.78	0.78	0.79	0.81	0.82	0.77	0.81	0.82	0.8	0.81	0.8
**KNN**	0.79	0.81	0.84	0.8	0.79	0.82	0.8	0.78	0.8	0.79	0.81	0.8	0.78
**AdaBoost**	0.84	0.82	0.8	0.83	0.84	0.82	0.82	0.81	0.81	0.83	0.81	0.82	0.78
**XGBoost**	0.85	0.84	0.82	0.85	0.86	0.84	0.84	0.82	0.85	0.84	0.86	0.81	0.83
**Extratrees**	0.82	0.83	0.77	0.84	0.78	0.8	0.81	0.8	0.83	0.82	0.82	0.8	0.81
**Light GBM**	0.85	0.86	0.84	0.88	0.86	0.85	0.83	0.82	0.84	0.86	0.86	0.84	0.82
**CatBoost**	0.85	0.85	0.81	0.84	0.81	0.82	0.83	0.82	0.85	0.84	0.83	0.85	0.78
**STACK-1**	0.81	0.83	0.83	0.85	0.81	0.82	0.8	0.83	0.8	0.81	0.81	0.82	0.81
**STACK-2**	0.84	0.87	0.86	0.89	0.85	0.84	0.85	0.85	0.85	0.88	0.86	0.82	0.83
**STACK-3**	0.85	0.86	0.84	0.89	0.85	0.84	0.84	0.84	0.8	0.86	0.87	0.83	0.81

As observed in Table 3, the forward feature selection method had a drastic range of accuracies across different models. The lowest accuracy in the table was of 0.72 obtained by SVM Sigmoid, whereas highest accuracy (0.89) across all pipelines was for the STACK architectures with FFS. Table 4 depicts the hyperparameters tuned by the GridSearchCV for the classifiers trained with FFS data. The optimum parameters helped attain the best performance.

**Table 4:** Selected hyperparameters for ML classifiers by GridSearchCV for models trained by FFS engineered data.

Machine Learning Classifier	Best Parameter Specifications
Logistic Regression	{'C': 0.01, 'penalty': 'l2'}
Decision Tree	{'criterion': 'gini', 'max_depth': 9, 'max_features': 'auto', 'min_samples_leaf': 11, 'min_samples_split': 50, 'splitter': 'best'}
Random Forest	{'bootstrap': True, 'max_depth': 90, 'max_features': 2, 'min_samples_leaf': 3, 'min_samples_split': 8, 'n_estimators': 300}
Support Vector Machine- linear kernel	{‘gamma’ = ’auto’, ‘kernel’ = ’linear’}
SVM-Polynomial kernel	{‘kernel’ = ’poly’, ‘max_iter’ = 200}
SVM-Gaussian kernel	{‘kernel’ = ’rbf’, ‘max_iter’ = 100}
SVM-Sigmoidal kernel	{‘kernel’ = ’sigmoid’, ‘max_iter’ = 1700}
Naïve bayes	{'var_smoothing': 10}
K-Nearest Neighbors	{'n_neighbors': 6}
AdaBoost	{'learning_rate': 0.1, 'n_estimators': 1000}
XGBoost	{'colsample_bytree': 0.3, 'gamma': 0.2, 'learning_rate': 0.15, 'max_depth': 8, 'min_child_weight': 1}
Extratrees	{'min_samples_leaf': 25, 'min_samples_split': 25, 'n_estimators': 50}
Light GBM	{'lambda_l1': 1.5, 'lambda_l2': 0, 'min_data_in_leaf': 30, 'num_leaves': 31, 'reg_alpha': 0.1}
CatBoost	{'border_count': 10, 'depth': 3, 'iterations': 250, 'l2_leaf_reg': 1, 'learning_rate': 0.03}

**Fig. 8:**
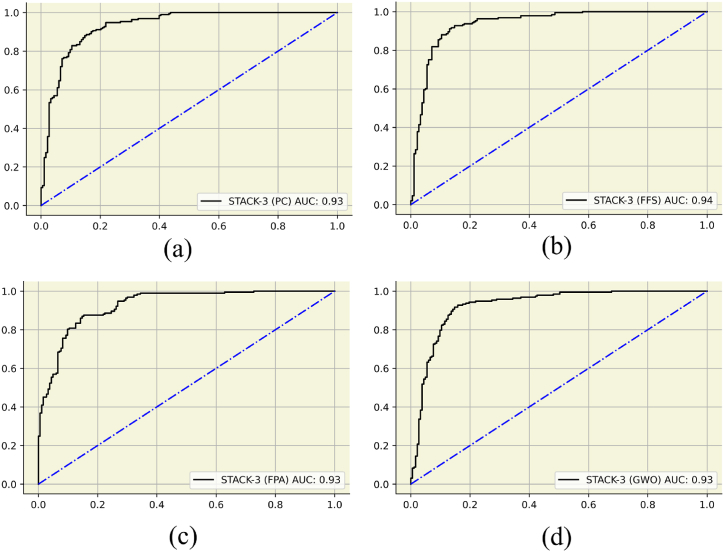
AUC plots for the best performing STACK-3 models. (a)STACK-3 trained on PC engineered data, (b) STACK-3 with FFS data, (c) STACK-3 with FPA data, (d) STACK-3 trained with GWO engineered data.

Regardless of the feature engineering technique, the STACK-3 model was the most robust classifier among all other sub-classifiers. Table 5 depicts the performance metrics for STACK-3 trained by PC, FFS, FPA, and GWO data. The results of the above four stacks were tabulated since they achieved excellent results. STACK-3 trained by FFS data had the best results with precision, recall, and Mathew's correlation coefficient of 88 %, 90 %, and 0.77, respectively. FFA-STACK-3 pipeline had the lowest hamming loss and log loss of 0.11 and 3.95, respectively. The STACK-3 GWO pipeline scored higher than the PC and FFS pipeline STACK-3 classifiers. More insights into STACK-3 performance based on the AUC-ROC curve can be observed in Fig. 8. FFS with STACK-3 pipeline has the highest score of 0.94 in Fig. 8(b), while the others have AUC-ROC of scores of 0.93 observed in Fig. 8(a), (c) and 8(d). Higher AUC scores correspond to a more reliable and high-performing classifier.

**Table 5:** Performance metrics for the best performing STACK-3 pipeline.

Models	STACK-3 (PC)	STACK-3 (FFS)	STACK-3 (FPA)	STACK-3 (GWO)
Performance Metrics				
Accuracy	0.86	0.89	0.86	0.87
Precision	0.87	0.88	0.85	0.88
Recall	0.87	0.90	0.88	0.88
F1-score	0.87	0.89	0.86	0.88
AUC-ROC score	0.93	0.94	0.93	0.93
Hamming Loss	0.14	0.11	0.14	0.13
Jaccard score	0.76	0.80	0.76	0.78
Log loss	4.78	3.95	4.96	4.41
Mathew's Correlation Coefficient	0.72	0.77	0.71	0.74

### 3.1 Explainable artificial intelligence to interpret classifier predictions

This sub-section provides a comprehensive analysis of the applications of XAI techniques to provide the risk of a patient having osteoporosis. Most XAI models do not process the stacked models due to a lack of support with the StackingClassifier function. Hence, we have deployed tools like SHAP, LIME, ELI5, and Qlattice and feature importance on our best tree-based pipelines.

#### 3.1.1 SHapley additive exPlanations (SHAP)

SHAP is a game theory-based explainable technique that is widely used in the domain of healthcare AI **Chadaga et al**. [48]. This technique produces SHAP values for all parameters based on the individual contribution to the target variable. Below are various SHAP visualizations that explain the Light GBM model (trained on FFS-engineered data). This pipeline's accuracy, precision, and recall were 88 %, 87 %, and 89 %, respectively. SHAP can give both local and global prediction explanations.

**Fig. 9:**
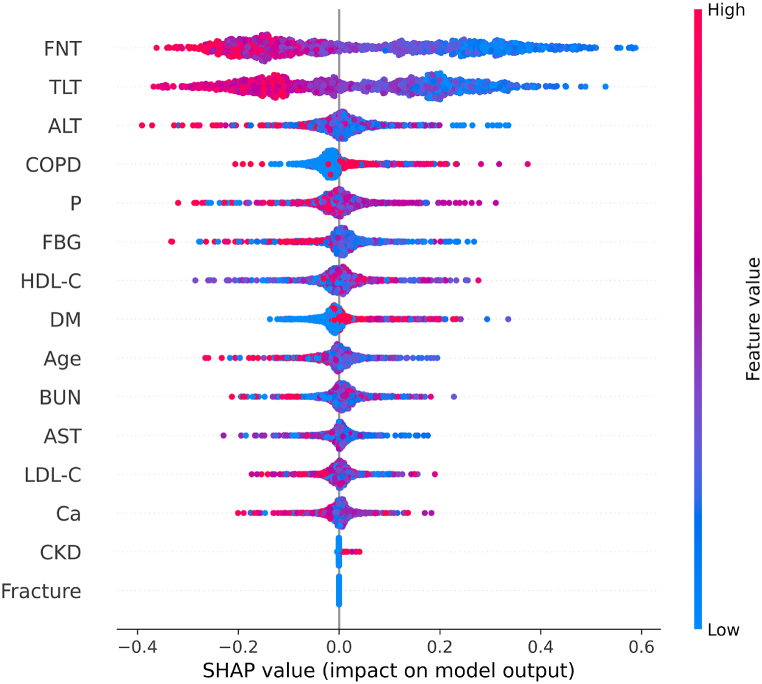
Shap Beeswarm plot.

• **SHAP Beeswarm plot:** This plot provides an information-dense summary of a feature's impact on the target variable. The features are ranked top to bottom based on SHAP mean. The feature on top has the most significant contribution to a prediction. Each dot on the plot represents an instance. A red dot indicates a higher feature value, and a blue dot indicates a lower value. The Beeswarm plot for our dataset can be seen in Fig. 9. Features like FNT and TLT scores are of the most significance. These two features have an opposite relation with the SHAP values. An increase in feature value causes a decrease in the SHAP values. This illustrates that a lower T-score would increase a patient's risk of Osteoporosis. COPD has a positive relationship with the SHAP values, indicating that a COPD patient can be at risk of Osteoporosis.

**Fig. 10:**
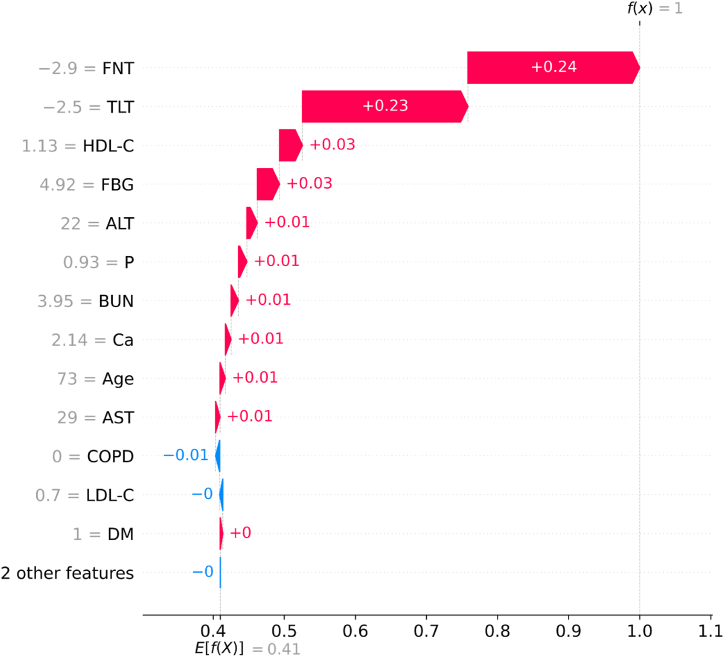
SHAP Waterfall plot.

**Fig. 11:**
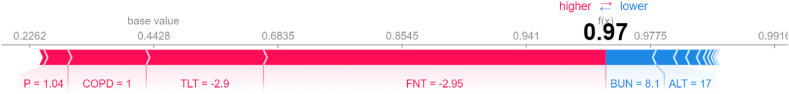
SHAP Force plot.

**Fig. 12:**
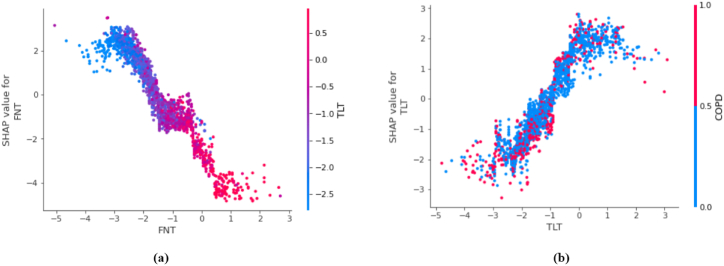
SHAP Dependence plots: (a) Indicates the interaction between FNT and TLT values, (b) Indicates the association between the TLT scores and presence of COPD.

• **SHAP Waterfall plot:** The SHAP waterfall plots display explanations for an individual patient. X-axis indicates the expected value of the classifier output. Each row indicates the contribution of each feature by the shift from the expected value. Red and blue signify the positive and negative shifts, respectively. The features along the Y-axis have individual parameter readings in grey. As observed in Fig. 10, features like FNT and TLT having less than or equal to −2.5 T-score have primarily contributed to the patient being diagnosed as OP-positive. In the third row, the HDL-C value is less than the healthy level. ALT, P, and BUN levels have a minor yet positive impact on osteoporosis prediction. The phosphorous level of this patient is severely less than normal. This waterfall plot is for a patient predicted OP-positive by the Light GBM classifier. Our classifier interpretation agrees with the ongoing research on osteoporosis (Rachner, Khosla, and Hofbauer [4]).

• **SHAP Force plot:** The plot in Fig. 11 provides a local-interpretation visual of SHAP values with an additive force layout. Similar to the plots mentioned above, this allows the user to observe individual feature contributions while the classifier makes a prediction for one instance (patient). The model's score for this patient is 0.97. Higher scores lead to a prediction of 1 (OP-positive prediction). The features are categorized into red and blue, indicating their contributions to pushing the score higher or lower. The features that influence the prediction are placed closer to the dividing boundary. The size of the bar for a feature is proportional to its impact on the Light GBM prediction. In Fig. 11, FNT and TLT scores are lower than −2.5. The patient suffers from COPD and has lower phosphorous levels. All these features in red have increased the model score by close to 1. The force plot agrees with the medical research, and this patient is at higher risk of OP (Leidig-Bruckner, G., and R. Ziegler. [5]).

• **SHAP Dependence plot:** The dependence plot shown in Fig. 12 depicts the interaction between two features. The X-axis shows the feature's values, and the Y-axis indicates that feature's SHAP value. The colors correspond to the second feature. Each point on the plot represents a patient. If an interaction is, observed, vertical patterns can be observed. In Fig. 12 (a) indicates the relation between the T-scores of the Femoral Neck and Thoracolumbar areas. A lower TLT score corresponds with a lower FNT value, and an increase in the TLT score increases the FNT value. The detection of osteoporosis is done in multiple skeletal areas. For patients in this study, the reduced t-score in the FN area was related to the reduced density in the TL area. In Fig. 12 (b) most patients facing COPD had a lower T-score indicating an association of COPD increasing the risk of osteoporosis.

#### 3.1.2 Local interpretable model-agnostic explanations

**Fig. 13:**
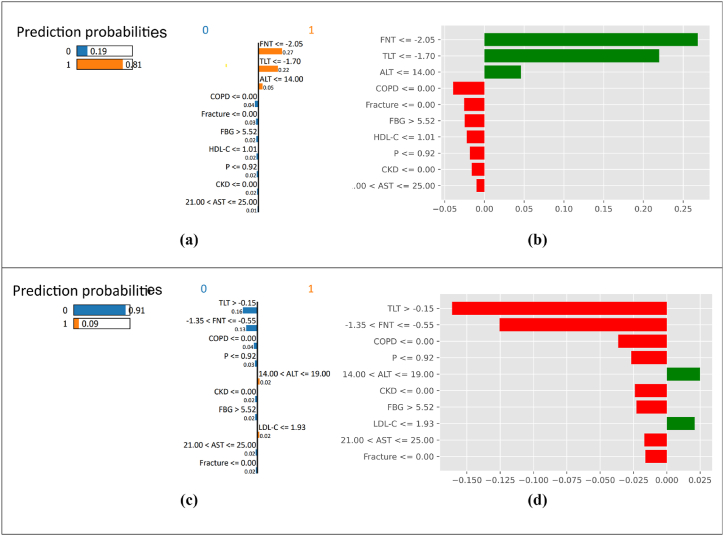
LIME plot: (a) Prediction probability chart for a patient-1 at high risk of OP, (b) Individual parameter contribution plot of patient-1. (c) Prediction probability chart for patient-2 at low risk of OP, (d) Individual parameter contribution plot of patient-2.

LIME makes a classifier interpretable by approximating the local patient-wise interpretations. In this study, we have explained. The Random Forest (trained with FFS data) pipeline achieved high accuracy, precision, and recall metrics of 88 %, 86 %, and 90 %, respectively. Hence, we deployed LIME to interpret RF-FFS architecture. LIME perturbs the input data to understand its effect on the predictions made by the classifier. The output of this model-agnostic XAI technique indicates the contribution of individual features to the prediction made by the RF-FFS classifier. The parameters with orange rows indicate a positive contribution to classifications, and the blue rows correspond to the negatively contributing features. Fig. 13(a) and 13(b) correspond to patient 1. This patient is at a higher risk of OP. Plot (b) depicts the ggplot (grammar of the graphics plot). As shown in Fig. 13(b), green and red bars represent the features and their contributions. The size of the bar indicates its contribution to the output. Green and red bars represent the shift indicating the positive or negative impact on the prediction. In Fig. 13(a) and (b), it is observed that TLT and FNT values were significantly low, wherein it led to a high risk of OP classifier prediction with a probability of 0.81. In Fig. 13(c) and (d), the patient had normal TLT, FNT, and phosphorous values. The patient did not suffer from COPD, significantly lowering the OP risk to a 0.09 probability. This risk prediction of OP aligns with the detection of OP (Rachner, Khosla, and Hofbauer [4]).

#### 3.1.3 ELI5

This tool is a python package that assists in the debugging of complex machine-learning models and provides an explanation for the predictions made. ELI5 can produce global and local interpretations **Chadaga et al**. [48]. In this study, we deployed ELI5 for the random forest algorithm (trained with FFS data). Table 6(a) depicts the weight assigned to the features based on the calculation of the gini index. FNT has the largest weight, and this operates as the root node. Table 6(b) indicates an individual prediction of a patient at high risk of OP (0.905 probability). The values of FNT and TLT are significantly low. The patient in consideration suffers from COPD. The parameter values and contributions have led to a prediction of 1.

**Table 6:**
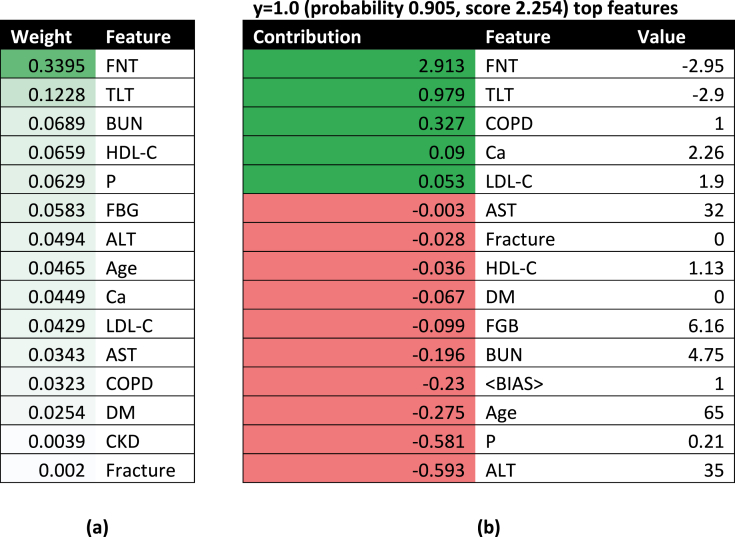
a) Depicts the feature weights and Table (b) Indicates individual patient-wise prediction.

#### 3.1.4 Qlattice

**Fig. 14:**
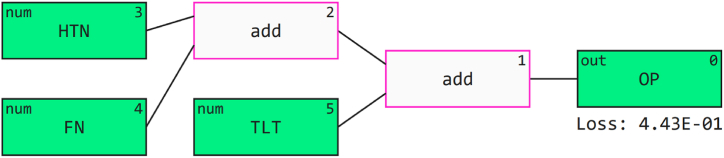
QGraph.

Qlattice is a software library that provides a python-based architecture that searches through all potential models and selects the model with the optimal features to provide a perfect fit for the data. This tool operates by a supervised learning approach. The feyn library provides a Qlattice model creation. Qlattice can process categorical and numeric data. Further, this tool allows the user to generate plots and evaluate the mathematical equations that potentially explain the model fit and design **Chadaga et al**. [48]. Fig. 14 depicts QGraphs providing insights into the features and operators considered while building a model. Equation (11) indicates a simplified equation for the model formed. Where FN, HTN and TLT are input independent features. The three input variables were considered and a model was built on the relationship between these variables to produce an output prediction.

(11) logreg(−7.76*(FN)+0.186*(HTN)−1.05*(TLT)+4.77)

**Fig. 15:**
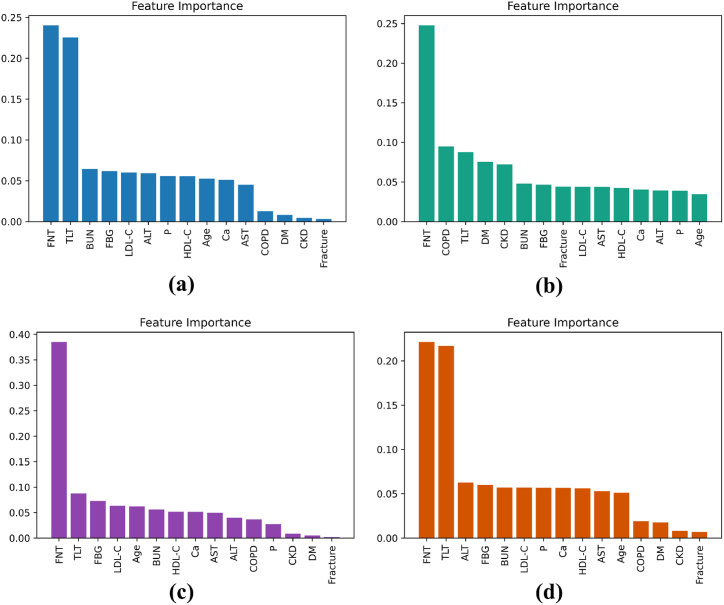
Feature Importance (FI): (a) With Random Forest, (b) With XGBoost, (c) With Decision Trees and (d) With Extratrees classifier.

Further, feature importance was used to compare the interpretations made by the above XAI techniques. In this study, we have deployed feature importance for Random Forest, XGBoost, Decision trees, and Extratrees trained on FFS-engineered data **Chadaga et al**. [48]. The scores for each feature in tree-based models are based on the decreasing node impurity weighted by the probability of reaching the node. Fig. 15 has four descending plots describing the feature importance of various tree-based classifiers. FNT was considered the most significant parameter in OP risk prediction in all four plots 15(a), 15(b),15(c) and 15(d). Fig. 15(a), (c), and 15(d) estimated TLT to be the second most important feature. Fig. 15 indicates that apart from the initial significant features, the importance score reduces drastically from left to right. Among the deployed tree-based models, RF had the highest score, and the feature importance ranking provided by RF supplements the research on risk prediction of OP (Rachner, Khosla, and Hofbauer [4]). Both the feature importance and XAI have suggested features of T-scores of FNT and TLT along with ALT and HDL-C levels, and the presence of COPD and HTN contribute towards the risk prediction of OP. Lower than −2.5 T-scores, higher HDL-C levels presence of cardiovascular disorders contribute towards increasing the risk of OP. ALT levels are known to have a negative relation with bone mineral density parameters (Leidig-Bruckner, G., and R. Ziegler. [1]). It is observed that the previously discussed XAI results, and feature importance results are majorly similar. This further establishes the reliability of such XAI tools.

Multiple research projects have been published on constructing Machine Learning models trained on tabular data for predicting the risk of osteoporosis. Primarily DXA scans and T-scores are used to train such models. However, there are multiple holistic risk prediction and detection algorithms. We will further compare the performance of these architectures with our best-performing pipelines. **Nam et al**. [51] analyzed data from 70 patients that underwent quantitative Computer-Tomography (CT) and conventional lumbar Computer-Tomography. They deployed a multiple regression algorithm to predict T-score primarily using parameters such as age, gender, Hounsfield units, and conventional CT along with the Quantitative CT. A logistic regression classifier produced an accuracy of 92.5 %. With a precision and recall of 93.9 % and 96.69 %, respectively. The risk of major osteoporotic fractures (consisting of fractures of the hip, wrist, spine, and humerus) was predicted by a machine-learning approach proposed by **De Vries et al.** [52]. Data from 7578 patients above the age of 50 years were considered for this study. Cox regression, Random Survival Forests, and Artificial Neural Networks were deployed. The highest concordance index of 0.697 was obtained for Cox regression. Hence, they built a risk calculator with Cox regression for osteoporosis and osteopenia patients. **Ordóñez et al** [53] proposed a machine learning based model for post-menopausal women. Parameters related to bone mineral density, lifestyle, and diet for 305 post-menopausal women were considered. Classifiers such as SVM and regression trees were considered. SVM outperformed the regression trees with a testing error of 2.05.7e-004.

Most studies primarily focus on bone density parameters for OP risk prediction. However, multiple other factors like glucose levels, lipid profiles, hepatic panels, and the presence of various cardiovascular disorders can increase the risk of OP. Selecting these significant parameters is crucial in creating a reliable prediction model. We created several pipelines with 13 feature selection techniques to ensure the best features are selected. Providing a comprehensive comparison among different pipelines helped understand individual performance of such pipelines. Further, the twelve machine learning classifiers were trained under each set of architectures. We employed a multi-level stacking approach to obtain the most robust and reliable architecture for predicting the risk of osteoporosis. We deployed four explainable AI techniques to make the pipeline outputs meaningful and interpretable. Despite the limited data, this study has the potential to be expanded by using more feature selection techniques and ML algorithms to conduct a more comprehensive analysis.

In the future, we aim to analyze the importance of advanced optimization algorithms, such as hybrid heuristics, metaheuristics, adaptive algorithms, self-adaptive algorithms, island algorithms, polyploid algorithmsRed deer and Social Engineering algorithms, and hyperheuristics, can be used for solving challenging decision problems **(Chen and Tan** [54]**, Dulebents et al.** [55]**, Pasha et al.** [56]**, Dulebents et al.** [57] **and Singh and Pillay** [58]**).**

## 4 Conclusion

Osteoporosis is a prevalent public health issue. This disorder is asymptomatic, eventually manifesting as bone fractures due to weakened bone structures. Predicting the risk of OP by holistic consideration of various blood, lifestyle, and bone mineral density parameters could assist in the patient seeking the required treatment before complete bone degradation. This study evaluated combinations of 13 feature selection techniques and 12 machine learning classifiers. The best-performing architecture was the multi-level STACK-3 trained by the Forward Feature Selection engineered data. Further, we deployed XAI techniques such as SHAP, LIME, ELI5, and Qlattice to demystify the machine-learning models. With this research, we observed that the bone mineral density parameter proved to be the most significant feature. However, features indicating lipid profiles, hepatic-panel readings, and prevalence of various disorders were found to influence the risk of osteoporosis.

Such architectures can be merged with the electronic health record interface. A patient is often unaware of osteoporosis because of its silent characteristics. In these situations, a risk prediction system could process various parameters and assist the patient in acquiring the appropriate medical treatment. However, such systems require high-quality, unbiased data and it is essential to conduct extensive medical validation and testing before deployment in healthcare facilities. Medical Decision Supports can improve efficiency and provide optimization of resources and time for healthcare providers. These models can be implemented as a dynamic system as it can constantly update parameters to detect new patterns and trends in the data. Such models have a potential of contributing to the sustainable development goals. While translating informatics into medical diagnosis, it is essential to have a layer of transparency to establish a strengthened association between the medical and informatics domains.

## Disclosure of interest

No potential competing interest was reported by the authors.

## Funding

No funding was received.

## Ethical clearance

Not applicable.

## Data availability statement

Data will be made available on request.

## CRediT authorship contribution statement

**Varada Vivek Khanna:** Writing – original draft. **Krishnaraj Chadaga:** Writing – review & editing, Resources. **Niranjana Sampathila:** Writing – review & editing, Supervision. **Rajagopala Chadaga:** Visualization, Investigation. **Srikanth Prabhu:** Conceptualization. **Swathi K S:** Writing – review & editing. **Aditya S. Jagdale:** Validation. **Devadas Bhat:** Software, Resources.

## Declaration of competing interest

The authors declare that they have no known competing financial interests or personal relationships that could have appeared to influence the work reported in this paper.
